# The treatment strategies for spine fractures in patients with ankylosing spondylitis: A case report

**DOI:** 10.1097/MD.0000000000008462

**Published:** 2017-11-03

**Authors:** RuSen Zhu, WenYe Song, Wei Hu, ZeHua Jiang, JianJun Yuan, ZiJian Cui, Jun Wan, Yan Liu, ShiQing Feng, XueLi Zhang

**Affiliations:** a Department of Spine Surgery, Tianjin Union Medicine Centre; b Department of Orthopedics, Tianjin Medical University General Hospital, Tianjin, PR China.

**Keywords:** ankylosing spondylitis, bone grafting, posterior instrumentation, spine fracture, surgery

## Abstract

**Purpose:**

The aim of the study was to explore surgical strategies for effectively treating spine fractures in patients with ankylosing spondylitis (AS) and investigate the postoperative outcomes.

**Methods:**

We retrospectively analyzed 9 patients with AS that was complicated by spine fractures, who underwent surgery at our spine and spinal cord clinic between 2005 and 2012. The surgical methods included posterior instrumentation and bone grafting, with or without decompression. Fracture healing and neurological function recovery were observed postoperatively. The average follow-up period was 45.1 months (range: 24–60).

**Results:**

The 9 patients included 7 men and 2 women. Eight patients were treated with long posterior fixation. One patient underwent combined long posterior fixation and anterior bone grafting. The neurological function of these patients improved significantly to varying degrees after surgery, and fracture union and segmental instability of the lumbar vertebrae were radiologically confirmed at the 1-month and 2-year follow-up intervals. Severe surgery-related complications were not observed.

**Conclusions:**

Posterior instrumentation with bone grafting was a satisfying method for treating spine fractures in patients with AS.

## 1 Introduction

Ankylosing spondylitis (AS) is a progressive, inflammatory rheumatic disorder that is predominant in men. In patients with AS, the axial skeleton and sacroiliac joints are exposed to syndesmophytosis and osteoporosis, especially in those with long-standing AS. AS eventually leads to a rigid and brittle bamboo-like spine, which is a characteristic of the disease that is seen on imaging.^[1,2]^ Osteopenia and osteoporosis, which develop early in the course of AS, have been found in the spine and hips of patients with this disease by measuring their bone mineral density.^[3,4]^ The prevalence of osteoporosis in patients with AS has been reported to be between 18.7% and 62%.^[5]^ Osteoporosis in patients with AS contributes to uncoupling of the bone formation and bone resorption processes, leading to spinal fracture after minor trauma. It even occurs in people without any trauma.^[6]^ The prevalence of osteoporosis is close to 25% after 10 years of disease, and vertebral fractures are seen in approximately 10% of patients with AS.^[7]^ This underlines the importance of effective and efficient surgical strategies for patients with AS that is complicated by spinal fracture.

Fractures in patients with AS are difficult to diagnose because the complaint of back or neck pain is often overlooked as a manifestation of AS. Standard imaging offers little help in diagnosing spine fractures in patients with AS because 2-dimensional images are often difficult to read, given the deteriorated anatomy that results from a kyphotic spine. The use of computed tomography (CT) and magnetic resonance imaging (MRI) is emphasized to provide more details about the fractured segments.^[8]^ Delayed diagnosis and improper nonsurgical treatment usually lead to pseudarthrosis, progressive neurologic deficits, and worsening deformity. In a retrospective study of a large, consecutive series of patients with spine fracture and ankylosing spinal disorders, the mortality rate for surgical patients (23%) was lower than that for nonsurgical patients (51%).^[9]^ This indicates that there was a higher rate of complications and mortality during nonsurgical treatment than during surgical intervention. However, surgical treatment can be fraught with complications. A multidisciplinary team approach with a thorough preoperative plan is essential for achieving good outcomes in AS patients at a high risk of developing fractures. The surgical approach depends on both the patient characteristics and the fracture's location and pattern.

We retrospectively studied clinically diagnosed spinal fractures in patients with AS who underwent surgery, to discover the most effective strategies for treating this disease.

## 2 Methods

This study has been approved by ethics committee of Tianjin Union Medicine Centre, and written consents were obtained from patients involved in the study.

We retrospectively analyzed 9 patients with AS complicated by spine fractures who underwent surgery at our spine and spinal cord clinic between 2005 and 2012. Seven of the patients were men and 2 were women, with a mean age of 44.6 years (range: 32–58 years). All patients fulfilled the modified New York criteria for primary AS. After hospital admission, all patients underwent a neuroimaging evaluation using plain radiography, CT scanning, and MRI. According to the American Spinal Injury Association (ASIA) grading system, the fractures of 5 patients were classified as class D, those of 3 were classified as class C, and that of 1 was classified as class B.

Eight patients were treated with long posterior fixation, and 1 patient underwent combined long posterior fixation and anterior bone grafting because the fracture site was extremely unstable. The follow-up duration was calculated from the date of admission to the most recent evaluation. The mean follow-up period was 45.1 months (range: 24–60). The clinical data of these patients are described in detail in Table 1.

**Table 1:**
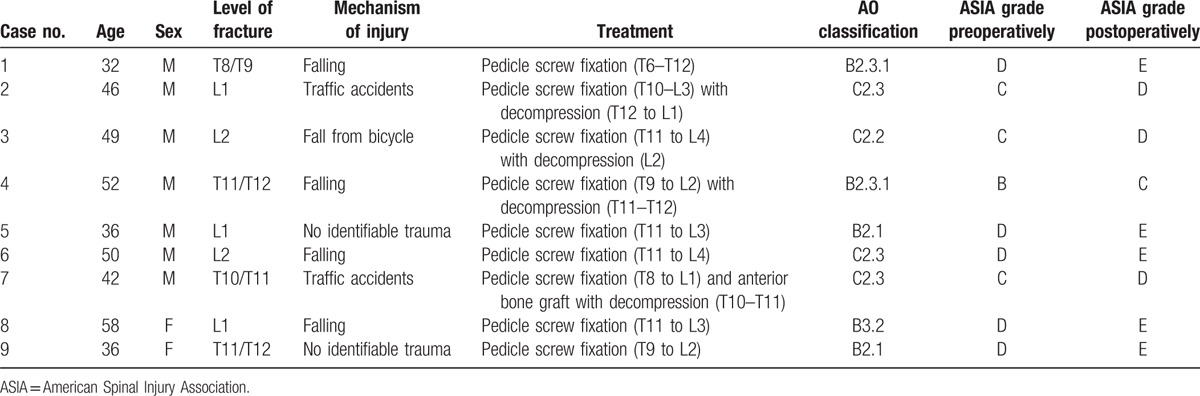
Clinical data and postoperative results of the patients in this study.

## 3 Results

The cause of injury included traffic accidents (2 patients), falling while walking or standing (4 patients), falling off a bicycle (1 patient), and unidentified trauma (2 patients). The fracture levels were T8 to T9 in 1 patient, T10 to T11 in 1, T11 to T12 in 2, L1 in 3, and L2 in 2 (Table 1). All patients had neurological deficits preoperatively. No patients experienced a total loss of motor and sensory function below the injured level. Three patients had motor weakness that was combined with a sensory deficit, and 6 patients had a sensory deficit.

The thoracic activity was restricted in AS patients with thoracolumbar fractures. Elderly patients might also have coronary ischemia, and young people may suffer from mild, limited respiratory function. In this study, the 9 patients were younger than those reported in the literature, with a mean age of 44.6 years (range: 32–58 years). Only 1 patient was older than 50. This 58-year-old patient had mild coronary ischemia, her preoperative indicators were normal after drug treatment, and no intraoperative and postoperative complications were observed. In the other 8 patients, no obvious cardiopulmonary dysfunction was observed; however, pneumonia was observed postoperatively in 1 patient, who was cured with antibiotic treatment.

Postoperatively, lower limb strength training and bowel and bladder function training were performed at a rehabilitation center. All patients were followed up with plain radiographs or CT.

After surgical treatment, the fractures healed in all patients, and their neurological function improved to varying degrees (Table 1). Surgery-related complications were not observed. No patients died during the follow-up period.

## 4 Typical case

A 46-year-old man presented with back pain and moderate paraparesis that was classified as ASIA grade C after experiencing a traffic accident. A physical examination revealed that the patient had thoracolumbar kyphosis and tenderness. He felt pain below the L1 level, and the saddle zone was weak. The muscle strength of the lower extremity was classified as grade 3/5. A CT scan revealed a burst fracture of the L1 vertebral body, with displacement of the spine. The patient was diagnosed with AS 10 years ago.

### 4.1 Surgical method

The patient was placed in a prone position after general anesthesia was administered. Conventional disinfection and draping were performed. A posterior longitudinal incision was made from the T9 to L4 level. The length of the incision was approximately 30 cm. The skin, subcutaneous tissue, and muscular fascia were cut layer-by-layer. After exposing the vertebral lamina, we could clearly see the fracture dislocation of T12 to L1. Posterior spinal fusion at T10 to L3 was performed utilizing 10 pedicular screws near the L1 level. A C-arm was used to determine the location of the pedicular screws. Laminectomy of T12 and L1 was performed for decompression. Laminar bone combined with allogenous bone was used for grafting between the transverse and articular processes from T10 to L3. Two lateral connecting rods were placed to complete fixation. Finally, a drainage tube was inserted and the incision was closed.

The operation time was 3 hours, and blood loss during surgery was 400 mL. This patient's plain radiographs and CT before and 2 years after the operation are shown in Figure 1 . At 2 years postoperatively, the fracture had healed, the ASIA grade had improved from C to D, and the muscle strength of the lower extremity had improved from grade 3/5 to grade 4+/5. The patient's bowel and bladder functions were normal postoperatively.

**Figure 1:**
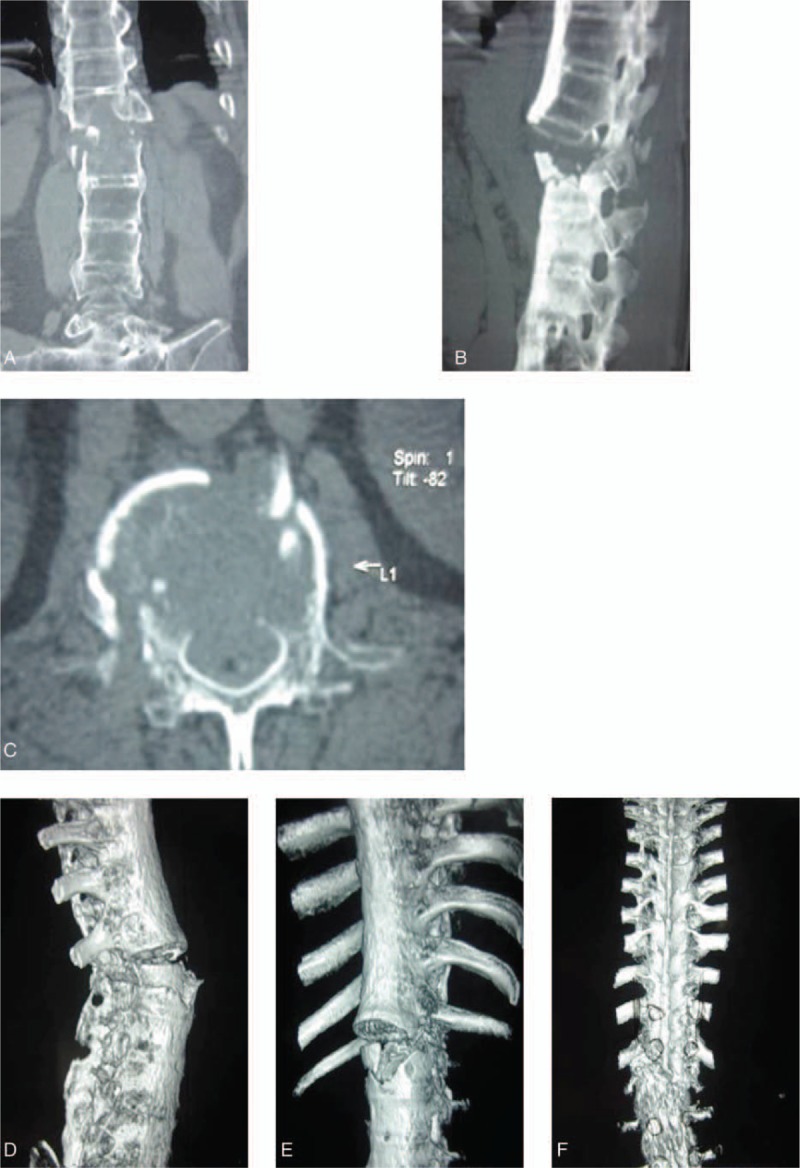
(A–C): Preoperative computed tomography (CT) coronal image (A), sagittal image (B), and axial image (C) revealed a burst fracture of the L1 vertebral body, with displacement of the spine. (D–F): Preoperative CT 3-D reconstruction images showing vertebral fracture in L1 and spinal rotation. (G) Preoperative magnetic resonance imaging (MRI) revealed vertebral fracture in L1 and spinal cord compression. (H–I): Posteroanterior (H) and lateral (I) X-ray projection 1 month after the operation revealed that fracture realignment and adequate stabilization were obtained. (J–K) Posteroanterior (J) and lateral (K) X-ray projection 2 years after the operation showing no further dislodgment of fracture with satisfactory bony healing. (L–O): CT 3-D reconstruction images revealed no loosening, breakage, and dislocation of the internal fixation system. Vertebral fracture was healed.

**Figure 1 (Continued):**
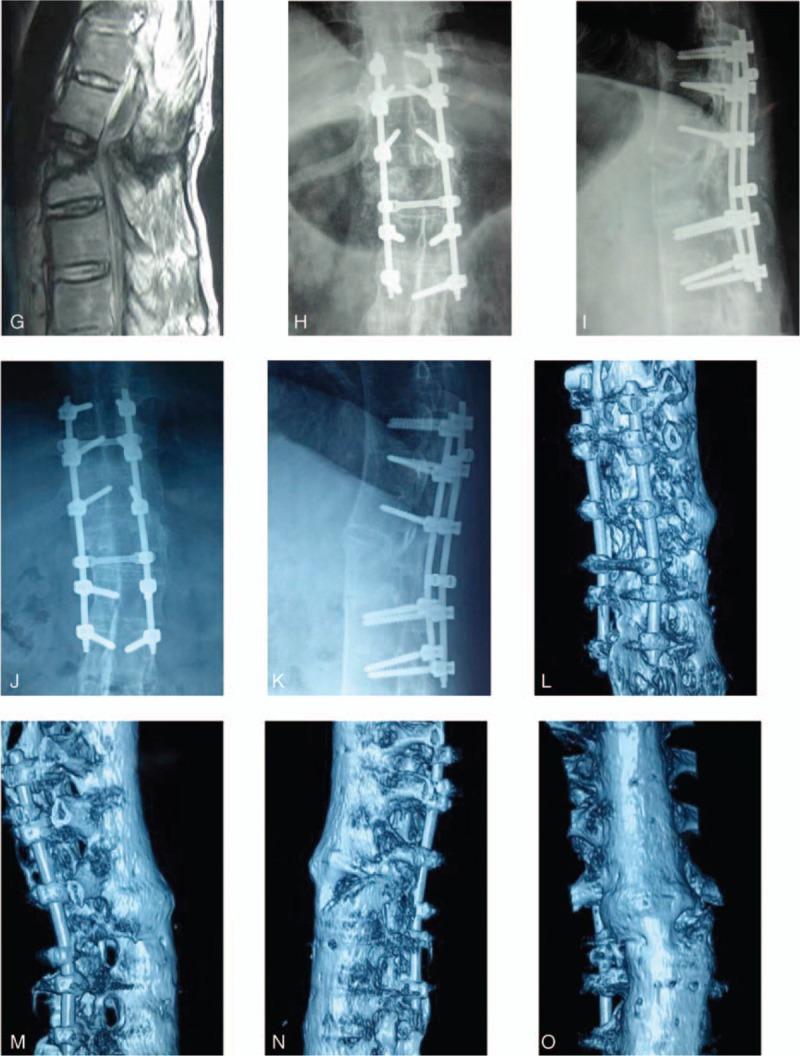
(A–C): Preoperative computed tomography (CT) coronal image (A), sagittal image (B), and axial image (C) revealed a burst fracture of the L1 vertebral body, with displacement of the spine. (D–F): Preoperative CT 3-D reconstruction images showing vertebral fracture in L1 and spinal rotation. (G) Preoperative magnetic resonance imaging (MRI) revealed vertebral fracture in L1 and spinal cord compression. (H–I): Posteroanterior (H) and lateral (I) X-ray projection 1 month after the operation revealed that fracture realignment and adequate stabilization were obtained. (J–K) Posteroanterior (J) and lateral (K) X-ray projection 2 years after the operation showing no further dislodgment of fracture with satisfactory bony healing. (L–O): CT 3-D reconstruction images revealed no loosening, breakage, and dislocation of the internal fixation system. Vertebral fracture was healed.

## 5 Discussion

Pathological remodeling of the spine in patients with AS involves 2 main features: inflammation and new bone formation.^[9]^ Inflammation results in intravertebral bone loss and bone erosion, and paravertebral and ligamentous ossification makes the entire spine similar to long and brittle bone. This places these patients at a high risk of developing a spinal fracture due to even trivial trauma.^[10]^ Studies indicate that vertebral fractures are a frequent finding in patients with AS, and their prevalence is higher in these patients than in those without AS.^[11]^ When spinal fractures appear in AS patients, diagnosis can be difficult, because fractures of the posterior arch are often difficult to visualize using conventional radiographs, and osteoporosis can further hamper the diagnosis. Both CT and MRI are usually recommended to provide more details about the fracture and spinal cord injury. CT scanning is routinely used to determine the fracture site, type, and severity. MRI scanning is used to determine tiny factures and spinal cord compression, especially when a neurological disability is observed. MRI is an essential and important method to determine whether decompression should be performed and the exact range of decompression. With either nonsurgical or surgical treatment, complications such as spinal cord lesions, nerve root lesions, and paravertebral hematoma are always more frequent and more serious in patients with AS than in those without AS, resulting in varying degrees of sensory or motor deficits. Therefore, in patients with severe or persisting neurological deficits, timely treatment (nonsurgical or surgical) is necessary. In an extensive meta-analysis, Westerveld et al^[12]^ noted that surgical treatment led to neurological improvement and a decreased overall complication rate compared with nonsurgical treatment at both short- and long-term follow-up. Nonsurgical treatment was performed only when surgical intervention would lead to unacceptably high perioperative risks due to a high rate of complications.

The primary aim of surgical treatment is to maintain fracture realignment, with adequate stabilization until the bone has healed completely.^[13]^ The main surgical methods include anterior stabilization, posterior stabilization, and a combined anterior–posterior surgical approach. Decompression of spinal stenosis may be performed in the same operative session to relieve neurological symptoms. The surgical approach depends on both the patient characteristics and the fracture's location and pattern. In one study, anterior stabilization alone failed to provide resistance against tension from the posterior column, resulting in loosening, pullout, or collapse of the bone graft and loosening or breakage of the internal fixators, which are associated with higher failure rates.^[14]^ Meanwhile, considering the prolonged operating time for double stabilization and greater bleeding volume, combined anterior–posterior surgical stabilization is not always possible. This is due to the higher risk of intra-operative and postoperative complications in stabilization than in a single anterior or posterior approach, let alone the cardiovascular and pulmonary problems that are caused by restrictive ankylosis and thoracolumbar kyphosis of the spine in patients with AS. This renders surgery extremely difficult for surgeons, especially when treating the cervical-thoracic junction. In clinical practice, these fractures are mostly managed with a single posterior approach using screw fixation via a formal open fusion procedure, which allows the surgeon to stabilize the injured segment, perform decompression with laminectomy, and recreate the preexisting alignment of the spine. No matter which kind of surgical method is chosen, it is important to preoperatively evaluate the fracture pattern, posterior ligamentous restraint, neurological status, preexisting deformity, and bone quality.^[15]^

Posterior stabilization is recommended for treating patients with unstable fractures who have a risk of translation to achieve a sufficiently stable anterior column.^[16]^ In this study, 8 patients were treated with long posterior fixation, and the treatment effect was satisfactory. The fractures healed in all patients, without screw loosening or backout and surgery-related complications. Only 1 patient underwent long posterior fixation and anterior bone grafting due to an anterior column defect. This patient also experienced prospective healing of the fracture and functional recovery.

Osteoporosis could be observed in most AS patients with thoracolumbar fractures. A rigid and brittle bamboo-like spine contributes to a greater lever arm. If only 2 or 3 vertebral segments are fixed, it is difficult to achieve firm fixation; meanwhile, greater stress concentration in a single segment during activity may lead to loosening, breakage, and dislocation of the internal fixation system. For most AS patients with spine fractures, vertebral foot pedicle screw fixation should be performed in 3 segments above and below the injured vertebral body to achieve good clinical efficacy. During the actual clinical treatment, 2 to 3 segments above and below the injured segment are usually chosen for long instrumentation and fusion, depending on the patient's circumstances and the various characteristics of the spinal fractures. This method can decrease the stress concentration in each segment and provide firm fixation to promote fracture healing and functional recovery.

With the help of CT and MRI scanning preoperatively, we selected segments for fixation and decompression. During posterior stabilization, one-time successful implantation of a pedicle screw and accurate pedicle positioning is the key to a successful operation. Using the guidance of a C-arm, it is easy to find the articular process accurately, remove part of the bone cortex, reveal the gap between the small joints, and locate the nailing point according to the transverse position. Therefore, blind nailing and spinal cord injury can be avoided, and sufficient fixation points can be achieved.

The clinical results of this study were consistent with those of other authors.^[17,18]^ Posterior stabilization was the most frequently used and efficacious method for treating spine fractures in AS patients. Combined 360° fusion is recommended for treating unstable fractures with translation or defects of the anterior column in patients with an additional kyphotic deformity. Laminectomy for decompression should be performed when compression symptoms are present.

In conclusion, patients with AS have a higher incidence of acute spinal fracture than the general population. These fractures are often unstable and require surgical treatment, which commonly includes posterior instrumentation and bone grafting. Decompression is also necessary when severe compression symptoms are present. Fracture healing and neurologic improvement were satisfactory in patients who underwent surgery in this study.

## Acknowledgments

We thank the patients for their time and effort and providing consent for our study.
