# The treatment strategies for spine fractures in patients with ankylosing spondylitis: A case report: Erratum

**DOI:** 10.1097/MD.0000000000009298

**Published:** 2017-12-15

In the article, “The treatment strategies for spine fractures in patients with ankylosing spondylitis: A case report”,^[1]^ which appeared in Volume 96, Issue 44 of *Medicine*, affiliation a should appear as Department of Spine Surgery, Tianjin Union Medical Center.

The authors would like it noted that RuSen Zhu and WenYe Song contributed equally to the article and are co-first authors.

The authors would like it noted that the research was funded by the State Key Program of National Natural Science Foundation of China (81330042); the Special Program for Sino-Russian Joint Research Sponsored by the Ministry of Science and Technology, China (2014DFR31210); the International Cooperation Program of National Natural Science Foundation of China (81620108018); the Key Program Sponsored by the Tianjin Science and Technology Committee, China (14ZCZDSY00044, 13RCGFSY19000); and 2016 Tianjin health industry key research projects (16KG158).

## References

[1] Zhu R Song W Jiang Z The treatment strategies for spine fractures in patients with ankylosing spondylitis: A case report. *Medicine*. 2017 96:e8462. 29095296 10.1097/MD.0000000000008462 PMC5682815

